# Caspr2 antibodies in herpes simplex encephalitis: an extension of the spectrum of virus induced autoimmunity? – A case report

**DOI:** 10.1186/s12883-022-02637-x

**Published:** 2022-04-05

**Authors:** Klaus Berek, Ronny Beer, Astrid Grams, Raimund Helbok, Anna Lindner, Bettina Pfausler, Alois Schiefecker, Florian Deisenhammer, Harald Hegen

**Affiliations:** 1 Department of Neurology, Medical University of Innsbruck, Anichstraße 35, 6020 Innsbruck, Austria; 2 Department of Neuroradiology, Medical University of Innsbruck, Innsbruck, Austria

**Keywords:** Case report, Herpes simplex virus, Caspr2, VGKC, Autoimmunity

## Abstract

**Background:**

Post herpes simplex virus (HSV) autoimmune encephalitis has been reported mainly in association with NMDA receptor antibodies, however, never with Caspr2 antibodies.

**Case presentation:**

We report an 82-year old female patient with encephalitis who presented with aphasia, left temporo-mesial hyperintense lesion on MRI, epileptiform discharges on spot electroencephalography, cerebrospinal fluid (CSF) lymphocytic pleocytosis and who showed positive HSV polymerase chain reaction in CSF as well as antibodies against contactin-associated protein-like 2 (Caspr2).

**Conclusion:**

This is the first report of a patient with encephalitis who tested positive for HSV as well as for Caspr2 antibodies.

## Background

Herpes simplex virus (HSV) encephalitis is one of the most frequent causes of sporadic infectious encephalitis [1]. Patients usually present with fever, seizures, and altered mental status [2]. Delay in diagnosis and initiation of acyclovir treatment contributes to the observed high degree of morbidity and mortality [3]. Up to 25% of patients develop a secondary neurological decline after 3–4 weeks, which has recently been attributed to autoimmune encephalitis following HSV encephalitis [4, 5]. So far, antibodies against N-methyl-D-aspartate receptor (NMDAR) have been identified in these patients [4, 5]. Interaction of these antibodies with the NMDAR leads to cerebral dysfunction and a characteristic clinical syndrome [6]. Although there are several other neuronal surface proteins as targets in autoimmune encephalitis, e.g. leucine-rich glioma-inactivated 1 (LGI1), contactin-associated protein-like 2 (Caspr2), α-amino-3-hydroxy-5-methyl-4-isoxazolepropionic acid receptor (AMPAR) type 1/2 or gamma-aminobutyric acid-receptor (GABAR) type A/B which frequently cause limbic encephalitis [7], only cases of patients with HSV encephalitis and positive GABA_A_R antibodies have been reported apart from NMDAR antibodies [5, 8–11].

Here we present a female patient with encephalitis who was tested positive for HSV DNA in CSF as well as for Caspr2 antibodies in serum.

## Case presentation

An 82-year old woman with a pre-morbid modified Rankin Scale (mRS) score of 1 was referred to the emergency department due to confusion for two days. Besides breast cancer 41 years ago and herpes zoster skin infection three years ago, the patient suffered from pre-existing hypertension, hyperlipidemia, and hypothyroidism treated with doxazosin, statin, and levothyroxine. The initial clinical examination revealed apart from global aphasia no further neurological deficits. The patient was afebrile (with a body temperature of 36.6 °C).

**Fig. 1:**
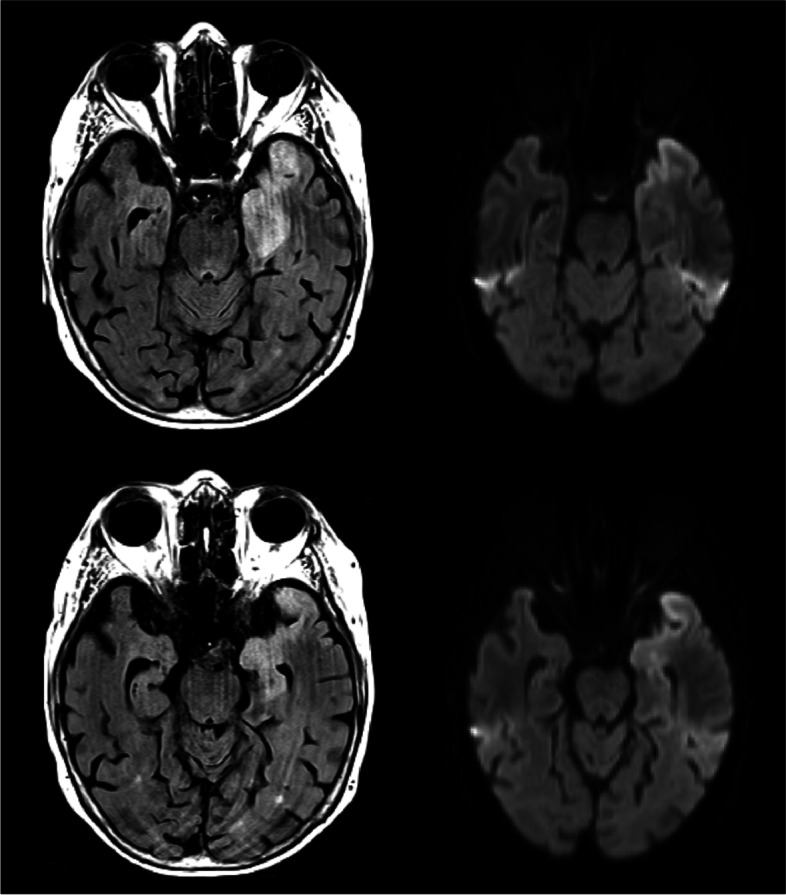
Brain MRI in a patient with encephalitis and positive HSV DNA and Caspr2 antibodies. Legend: T2-weighted MRI shows hyperintense lesion in the left temporo-mesial lobe (left) with diffusion restriction on the diffusion-weighted sequences (right)

Emergency cerebral MRI showed a T2-hyperintense swelling in the left temporo-mesial lobe, including cortical and subcortical areas, hippocampus and amygdala. Within these regions partially a diffusion-restriction of the cortex was found; in addition there was a thin contrast-enhancing rim subcortically at the lateral border of the T2-hyperintense swelling (Fig. 1). Electroencephalogram (EEG) revealed continuous bilateral slowing with periodic lateralized discharges. Cerebrospinal fluid (CSF) analysis at admission yielded a mild pleocytosis comprising mononuclear cells (white blood cell [WBC] count of 8/μl), a slightly disrupted blood-CSF-barrier (as indicated by elevated CSF total protein of 0.73 g/L and Q_alb_ of 11.3) and an intrathecal IgM synthesis (13%). HSV DNA was detected in CSF by means of polymerase chain reaction and diagnosis of HSV encephalitis was established. Acyclovir treatment (10 mg per kilogram bodyweight every 8 h) was started and the patient was transferred to the neurological intensive care unit. Antiepileptic treatment with Levetiracetam (2000 mg per day) was administered. An appropriate neuropsychological testing at this time was not feasible due to the global aphasia (Mini-Mental State Examination Score [MMSE]: 3).

**Fig. 2:**
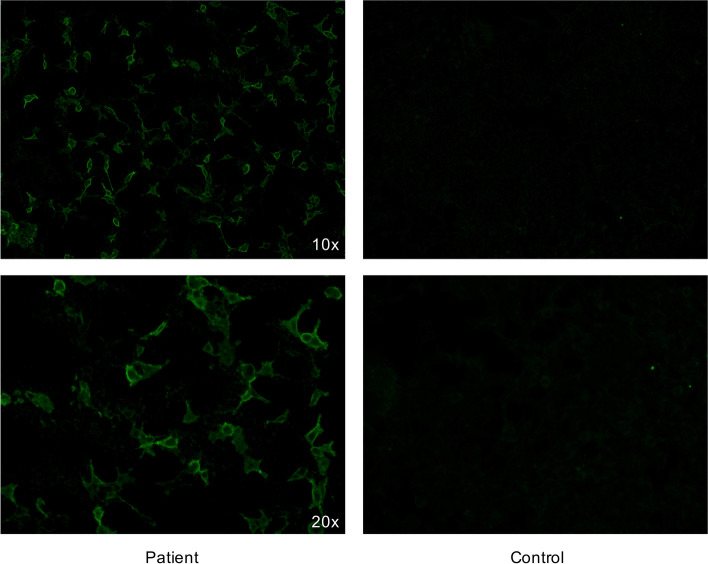
Detection of Caspr2-antibodies by immunofluorescence. Legend: Presence of Caspr2 antibodies was determined by a cell-based immunofluorescence assay. Patient’s serum sample was added to fixed cells that express Caspr2 protein on their surface (left) as well as to non-transfected cells (right). Reactivity at a serum dilution of ≥ 1:100 proves Caspr2 antibodies. The immunofluorescence pattern is shown at a magnification of 10 x (above) and 20 x (below)

As HSV encephalitis might be associated with secondary autoimmunity [4, 5], we performed further autoimmunity work-up that revealed Caspr2 antibodies in serum (Fig. 2) but not in CSF using a commercially available cell-based assay (Euroimmun, Cat. Nr. FA1439–1005–1; Lübeck, Germany). Antibodies against NMDAR, LGI1, AMPAR type 1/2, GABA_B_R, immunoglobulin-like cell adhesion molecule 5 (IgLON5), dipeptidyl-peptidase–like protein 6 (DPPX) using a cell-based assay (Euroimmun), Yo, Hu, Ri, CV2, Ma2, Amphiphysin using an immunoblot (Euroimmun), aquaporin-4 (AQP-4) and myelin oligodendrocyte glycoprotein (MOG) using a cell-based assay as previously described [12] were not detected in both CSF and serum. A whole-body 18^F^-FDG PET/CT revealed no hypermetabolic active lesions, i.e. no evidence of tumor, especially no thymoma and no lung cancer were detected.

**Fig. 3:**
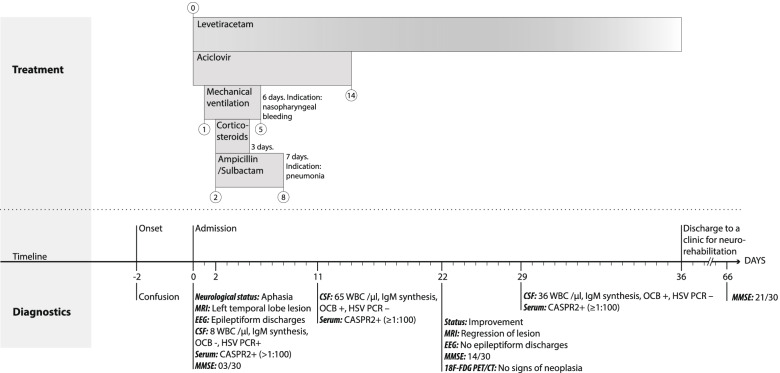
Essential diagnostic and therapeutic procedures. Legend: *Abbreviations*: Caspr2, contactin-associated protein-like 2; CSF, cerebrospinal fluid; EEG, electroencephalogram; MRI, magnetic resonance imaging; MMSE, Mini-Mental State Examination; F^18^-FDG-PET, 18-Fluoro-deoxyglucose positron emission tomography

Acyclovir treatment was maintained for 14 days and corticosteroids were administered additionally for three days. While the clinical course of the patient initially was mainly determined by complications (nasopharyngeal bleeding and subsequent mechanical ventilation, pneumonia), the patient started to improve thereafter. MRI was repeated after twenty-two days revealing a minor regression of the T2 hyperintense lesion in the left temporal lobe and a reduction of the left hippocampal volume. Neuropsychological testing showed a significant impairment with leading deficits in verbal fluency and memory performance (Consortium to Establish a Registry for Alzheimer’s Disease (CERAD) battery—semantic verbal fluency: 7; phonemic verbal fluency: 0; Boston Naming Test: 8; MMSE: 14). Epileptic discharges were no more detected by spot EEG. CSF analysis was repeated at day 11 and day 29. While WBC count first increased to 65/μl and eventually decreased to 36/μl, an intrathecal IgG synthesis (oligoclonal bands pattern II) occurred. HSV-PCR in CSF was negative at day 11 and 29. Presence of serum Caspr2-antibodies were repeatedly confirmed on day 11 and day 29. An overview on the main diagnostic procedures and treatments over time are shown in Fig. 3.

After approximately one month the patient was transferred for neuro-rehabilitation. At this time, she had a mRS score of 4. After neuro-rehabilitation, i.e. after another month, neuropsychological testing revealed further improvement, with ameliorated but still present deficits in memory performance and verbal fluency; the MMSE was 21. A clinical follow-up after 7 months revealed a mRS of 3, and no neurological deterioration occurred.

## Discussion and conclusions

Secondary brain autoimmunity develops in a subset of approximately 25% of patients with HSV encephalitis [4, 5]. The vast majority of these patients develop antibodies against NMDAR [4, 5], while only a few patients had antibodies against GABA_A_R, or antibodies against un-identified antigens [5, 8, 9, 11]. To the best of our knowledge this is the first report of a patient with encephalitis who tested positive for HSV DNA as well as for antibodies to Caspr2.

The present case shows similarities, but also some striking differences compared to previous reports. Patients with autoimmune post-HSV encephalitis pre-dominantly had a biphasic disease course and developed autoantibodies during follow-up before or at the clinical relapse (i.e. were antibody negative at the onset of HSV encephalitis) [4, 5]. Our patient did not experience any further neurological worsening during follow-up. It can be hypothesized that clinical symptoms, which might be similar between HSV encephalitis and post-HSV autoimmune syndrome, were indeed overlapping in our patient and – as previously reported – evolving in continuity [9]. Nevertheless, it seems more likely that the development of secondary autoimmunity as shown by the detection of Caspr2 antibodies remained clinically asymptomatic. A recent study that prospectively followed 51 HSV encephalitis patients reported that 50% of patients with detectable neuronal surface antibodies did not develop any clinical symptoms consistent with autoimmune encephalitis [5]. Besides the absence of a clinical deterioration during follow-up in our case (until to 7 months after onset), we want to clearly state that there were also no other typical signs and symptoms of Caspr2 autoimmunity such as peripheral nervous system hyperexcitability, dysautonomia or insomnia [7].

The timing of Caspr2 antibody positivity in our case remains elusive. To date, the development of secondary autoimmunity has been observed as early as seven days after HSV encephalitis (in a 2-month-old GABA_A_R positive post-HSV encephalitis patient) [8]. The overall disease duration of our patient before admission was about two days which would be a very short time for a de novo production of Caspr2 antibodies. Although the pre-existence of Caspr2 antibodies – i.e. prior to onset of HSV encephalitis– cannot be excluded, it seems unlikely, as our patient did not have any prior history of symptoms consistent with Caspr2 phenomenology [7].

Caspr2 antibodies in our patient were detected only in serum (three times, at a titer of ≥ 1:100), but not in CSF. In general, it is known that the prevalence of antibodies in immune-mediated neurological diseases is not fully congruent between the CSF and serum compartment. While there are certain diseases, e.g., NMDAR encephalitis, that show a higher frequency of autoantibodies in CSF than in serum [13–16], in other disorders, e.g. LGI1 [17, 18], GABA_A_R [8, 19], or AMPAR encephalitis [20], autoantibodies might be detected in certain cases only in serum (and not in CSF). Also, in neuromyelitis optica spectrum disorders or MOG associated disorders, both inflammatory diseases of the central nervous system, the diagnostic antibodies, i.e. AQP-4 and MOG antibodies, are mostly detectable in serum only [21–23].

There is some evidence that Caspr2 antibodies occur more frequently in serum in case of peripheral nervous system affection (i.e. Morvan syndrome, neuromyotonia) and more frequently in CSF in case of a central nervous system disorder (i.e. limbic encephalitis) [24, 25]. This might reflect different pathophysiological mechanisms. However, the ability to detect antibodies in the CSF or serum compartment is also most likely a matter of quantity. It has been clearly reported that Caspr2 titers are significantly lower in CSF than in serum [25].

The possibility of a false-positive Caspr2 antibody test result in our patient is very unlikely due to several reasons. First, Caspr2 antibodies were detected in three samples that were collected at different time points. Second, we used a cell-based assay that overall shows a good performance especially a high diagnostic specificity (at a dilution of ≥ 1:100) [26]. However, Caspr2 antibodies can be found in less than 1% of healthy individuals [27].

Here, we report a patient with encephalitis who was tested positive for HSV DNA in CSF and concomitant Caspr2 antibodies in serum. We conclude that Caspr2 antibodies reflects most likely an asymptomatic, secondary autoimmune phenomenon to HSV encephalitis. Whether this association indeed extends the spectrum of virus-induced secondary brain autoimmunity has to be further elaborated.

## Data Availability

The datasets used and/or analysed during the current study are available from the corresponding author on reasonable request.

## Abbreviations

AMPAR: α-Amino-3-hydroxy-5-methyl-4-isoxazolepropionic acid receptor
AQP-4: Aquaporin-4
Caspr2: Contactin-associated protein-like 2
CERAD: Consortium to Establish a Registry for Alzheimer’s Disease
CSF: Cerebrospinal fluid
DPPX: Dipeptidyl-peptidase–like protein 6
EEG: Electroencephalogram
GABAR: Gamma-aminobutyric acid-receptor
HSV: Herpes simplex virus
IgLON5: Immunoglobulin-like cell adhesion molecule 5
LGI1: Leucine-rich glioma-inactivated 1
MOG: Myelin oligodendrocyte glycoprotein
mRS: Modified Rankin Scale
NMDAR: N-methyl-D-aspartate receptor
WBC: White blood cell
